# Nanofabrication and Nanomanufacturing

**DOI:** 10.3390/nano12030458

**Published:** 2022-01-28

**Authors:** Mikhael Bechelany

**Affiliations:** Institut Européen des Membranes, IEM, UMR-5635, University Montpellier, ENSCM, CNRS, Place Eugène Bataillon, CEDEX 05, 34095 Montpellier, France; mikhael.bechelany@umontpellier.fr

Nanotechnology is a broad area integrating different research disciplines, including but not limited to material science, engineering, physics, chemistry, polymer science, optics, electronics, robotic, metallurgy, pharmacology, pharmacy and medicine. The Nanotechnology appellation has been used in different contexts: designing materials at the nanometric scale (synthesis, fabrication, etc.) and manipulation of nanomaterials and investigation properties at the nanodimensional level (mechanical, optical, electrical, etc. https://www.mdpi.com/2079-4991/12/2/177 (accessed on 30 November 2021)). Nanotechnology has found applications in different fields, such as food security, agriculture, medicine, energy, automotive, environmental protection, electronics, textiles and cosmetics.

The tuning of the morphology, size, porosity, organization, crystallinity and chemical composition of materials at the nanoscale, as well as their interfaces (surface charge, chemical function, hydrophobicity/hydrophilicity, etc.), is crucial to control their properties and allow their applications in various fields, such as electronics, photonics, energy, life sciences and the environment. The aim of this section is to focus on nanoprocessing approaches that allow us to create novel nanostructures and architecture by using innovative synthesis, fabrication and manufacturing methods, enabling the control of their properties, as well as their applications.

Research interests in the Section “Nanofabrication and Nanomanufacturing” include but are not limited to the following: synthesis, fabrication and manufacturing of nanostructured and nanoscale materials (bottom up and/or top down approaches); design of nanoparticles, quantum dots and clusters with control morphology and complex structures (core/shell, alloy, etc.); formation of 1D nanostructures (nanofibers, nanotubes, nanowires, nanorods, etc. https://www.sciencedirect.com/science/article/abs/pii/S2352940719305414 (accessed on 30 November 2021)); 2D materials and their heterostructures (graphene-based materials, silicate clays, layered double hydroxides, transition metal dichalcogenides, transition metal oxides, black phosphorus, hexagonal boron nitride, graphitic carbon nitride, etc.); coatings and thin films (Atomic Layer Deposition, Chemical Layer Deposition, etc. https://pubs.acs.org/doi/abs/10.1021/acs.chemmater.8b02687 (accessed on 30 November 2021)); hybrid nanostructures (organic/inorganic); nanocomposites; self-assembly and organization; nanostructured materials, such as Zeolites, MOFs and membranes (https://www.mdpi.com/2079-4991/9/11/1552 (accessed on 30 November 2021)); nanomaterial features tuned by the nanofabrication/nanomanufacturing approaches (catalytic activity; electrical, mechanical, magnetic and optical properties).

